# Detecting Subclinical Social Anxiety Using Physiological Data From a Wrist-Worn Wearable: Small-Scale Feasibility Study

**DOI:** 10.2196/32656

**Published:** 2021-10-07

**Authors:** Ruksana Shaukat-Jali, Nejra van Zalk, David Edward Boyle

**Affiliations:** 1 Dyson School of Design Engineering Imperial College London London United Kingdom

**Keywords:** social anxiety, wearable sensors, physiological measurement, machine learning, young adults, mental health, mHealth, new methods, anxiety, wearable, sensor, digital phenotyping, digital biomarkers

## Abstract

**Background:**

Subclinical (ie, threshold) social anxiety can greatly affect young people’s lives, but existing solutions appear inadequate considering its rising prevalence. Wearable sensors may provide a novel way to detect social anxiety and result in new opportunities for monitoring and treatment, which would be greatly beneficial for persons with social anxiety, society, and health care services. Nevertheless, indicators such as skin temperature measured by wrist-worn sensors have not been used in prior work on physiological social anxiety detection.

**Objective:**

This study aimed to investigate whether subclinical social anxiety in young adults can be detected using physiological data obtained from wearable sensors, including heart rate, skin temperature, and electrodermal activity (EDA).

**Methods:**

Young adults (N=12) with self-reported subclinical social anxiety (measured using the widely used self-reported version of the Liebowitz Social Anxiety Scale) participated in an impromptu speech task. Physiological data were collected using an E4 Empatica wearable device. Using the preprocessed data and following a supervised machine learning approach, various classification algorithms such as Support Vector Machine, Decision Tree, Random Forest, and K-Nearest Neighbours (KNN) were used to develop models for 3 different contexts. Models were trained to differentiate (1) between baseline and socially anxious states, (2) among baseline, anticipation anxiety, and reactive anxiety states, and (3) social anxiety among individuals with social anxiety of differing severity. The predictive capability of the singular modalities was also explored in each of the 3 supervised learning experiments. The generalizability of the developed models was evaluated using 10-fold cross-validation as a performance index.

**Results:**

With modalities combined, the developed models yielded accuracies between 97.54% and 99.48% when differentiating between baseline and socially anxious states. Models trained to differentiate among baseline, anticipation anxiety, and reactive anxiety states yielded accuracies between 95.18% and 98.10%. Furthermore, the models developed to differentiate between social anxiety experienced by individuals with anxiety of differing severity scores successfully classified with accuracies between 98.86% and 99.52%. Surprisingly, EDA was identified as the most effective singular modality when differentiating between baseline and social anxiety states, whereas ST was the most effective modality when differentiating anxiety among individuals with social anxiety of differing severity.

**Conclusions:**

The results indicate that it is possible to accurately detect social anxiety as well as distinguish between levels of severity in young adults by leveraging physiological data collected from wearable sensors.

## Introduction

### Background

Social anxiety is a fear of social situations in which the individual is exposed to possible scrutiny by others [1], and high levels of social anxiety are associated with a low quality of life in various domains [2,3]. Even when not clinically diagnosable (ie, subclinical or threshold social anxiety), it can greatly affect young people’s lives. Fehm et al [4] showed that young adults with social anxiety who do not receive treatment are at risk of developing social anxiety disorder (SAD) and comorbid mental health problems such as depression, both of which cause further adverse life impairments [3,5]. SAD is one of the most common anxiety disorders [6]. One UK study in 2000 [7] revealed that the annual health care cost per person with SAD was £609 (US $834.59), with annual productivity losses and social security benefits adding to £1920 (US $2631.22) per person with SAD, whereas those with SAD and a comorbidity incurred even higher costs. Nevertheless, many individuals do not receive treatment owing to limited availability or lack of awareness of social anxiety among health care professionals [3,4,8]. Some may not even seek treatment owing to a fear of being negatively evaluated by health care professionals [8]. Thus, it is imperative to empower both individuals and health care professionals in early detection of social anxiety before it potentially escalates into SAD and other related problems.

Common methods for assessing social anxiety involve using subjective measures, usually in a clinical setting. Owing to the rising prevalence of social anxiety, however, it is becoming evident that traditional approaches are inadequate and unsustainable for health care services [4,5,9]. In recent years, increasing focus has been given to technological advances that might help in the early detection and subsequent intervention for anxiety-related problems. In terms of social anxiety, objective methods used to assess symptoms include monitoring physiological changes typically caused by anxiety such as an elevated heart rate (HR), increased electrodermal activity (EDA), variation in skin temperature (ST), and trembling [1,10-12].

Nevertheless, despite extensive and promising research into stress and emotion detection based on physiological indices applicable to social anxiety collected from wearable sensors (Table 1), there has been little effort to predict social anxiety particularly using this approach. This might be ascribed to the recent shift in attention toward social anxiety reported by Heimberg and Butler [13], owing to widening of the diagnostic criteria and leading to a rise in those who fit the criteria for social anxiety.

Although not without its problems, detection via wearable sensors has the potential to underpin solutions addressing the growing needs of individuals with social anxiety and complement traditional therapeutic approaches. If subclinical social anxiety could reliably and validly be detected using wearable sensors, initial treatment could subsequently transition to digital self-help solutions to aid social anxiety at earlier stages when treatment is less extensive and costly [7]. Furthermore, self-help solutions may be a more appropriate method of treatment as individuals with social anxiety often feel nervous to seek treatment in clinical settings [8]. Detecting social anxiety using evidence-based objective methods could also complement current therapeutic approaches.

### Prior Work

#### Emotion Detection Using Machine Learning

A rise in wearable devices has further enabled researchers to investigate methods for the detection of emotion and stress states [14,15], with many studies reporting high-accuracy detection levels (Table 1). To detect emotional states using physiological data, researchers have executed data collection experiments that invoke the state to be detected, with tasks including hyperventilation and watching emotional films [16,17].

After data collection, a supervised machine learning (ML) approach is commonly used owing to the classification nature of the investigations [16-18]. In supervised ML, the training data are labeled in accordance with the correct class as the classification algorithms learn by example. Table 1 shows an overview of ML approaches focusing on emotion and stress detection. The most dominant and successful algorithm in studies involving recognition of states using physiological data is Support Vector Machine (SVM). Classifiers such as Decision Tree, Random Forest, and K-Nearest Neighbours (KNN) have also been frequently used and are reportedly effective.

**Table 1:** Studies on the recognition of emotion and stress states by using physiological indicators.

Study	Classification algorithms	Physiological data	Detection	Reported accuracy, %
[16]	SVM^a^, Decision Tree, KNN^b^, Naïve Bayes, Random Forest, Neural Network, Zero K	HR^c^, ST^d^, EDA^e^	Stress	65.8-100%
[18]	SVM	EDA, BVP^f^, PD^g^	Stress	57.1-80%
[19]	SVM, Decision Tree, KNN, Naïve Bayes	EDA, BVP, PZT^h^, EEG^i^, ECG^j^, EMG^k^	Emotion	17-91.3%
[20]	SVM	BVP, ST, EDA, PD	Stress	61.5-90.1%
[21]	KNN	HRV^l^	Stress	79.2-94.6%

^a^SVM: Support Vector Machine.

^b^KNN: K-Nearest Neighbours.

^c^HR: heart rate.

^d^ST: skin temperature.

^e^EDA: electrodermal activity.

^f^BVP: blood volume pulse.

^g^PD: pupillary distance.

^h^PZT: piezoelectric response.

^i^EEG: electroencephalogram.

^j^ECG: electrocardiogram.

^k^EMG: electromyography.

^l^HRV: heart rate variability.

#### Physiological Indicators of Social Anxiety

Classical psychological experiments commonly use impromptu public speaking tasks to elicit a social anxiety response [1,11,22,23]. These experiments are often split into stages that measure three responses: *baseline*, *anticipatory*, and *reactive* anxiety (where the nature of the speaking task is announced beforehand to provoke an anticipatory anxiety response) [1,11,23]. In conjunction, respondents are typically asked about their anxiety levels through self-reports [24]. Although self-reports are an important way of gauging individual perceptions of social anxiety, this approach is not without its problems, including a high level of subjectivity.

A more objective way to measure social anxiety is using physiological indicators. Social anxiety activates the sympathetic nervous system (SNS) [25]. HR and ST are modulated by both the parasympathetic nervous system (PNS) and SNS divisions of the autonomic nervous system (ANS), whereas EDA is modulated by the SNS alone. Therefore, EDA, HR, and ST are seen as markers of SNS activation and can be considered as potential indicators of social anxiety [26-29].

Studies investigating physiological responses to social anxiety further illustrate the potential to use EDA, HR, and ST as indicators [10-12]. Despite the potential for these indicators, however, their responses are complex, and a few studies have indicated minor differences in SNS arousal for individuals with social anxiety compared to control groups [22,23,30]. Furthermore, although ST has been explored as a social anxiety marker [12], wrist ST measurements have not been explored systematically. To our knowledge, this is the first study to explore wrist ST as an indicator of experimentally induced social anxiety.

### Research Aims and Objectives

This study aimed to investigate whether social anxiety in young people with subclinical social anxiety can be detected using physiological data (based on HR, ST, and EDA) recorded from an existing multi-sensor wearable. The study aims to explore if models can be trained to differentiate (1) between baseline and socially anxious states, (2) among baseline, anticipation anxiety, and reactive anxiety states, and (3) between social anxiety among individuals with social anxiety of differing severity. This study also aims to explore the predictive capability of the singular modalities.

## Methods

### Recruitment

**Figure 1:**
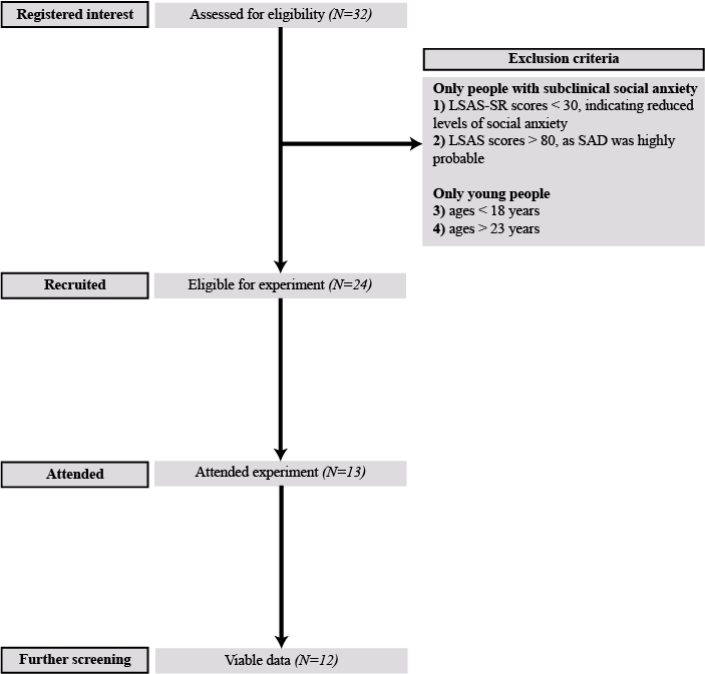
Flow diagram explaining the study recruitment process. LSAS-SR: self-reported version of the Liebowitz Social Anxiety Scale, SAD: social anxiety disorder.

Young adult participants were recruited using posters around Imperial College London. The initial sample comprised 13 individuals who self-identified as shy or socially fearful. An exclusion criterion was created to ensure that only young adults with subclinical social anxiety were recruited, as described in Figure 1. To assess participants’ social anxiety levels, the self-reported version of the Liebowitz Social Anxiety Scale (LSAS-SR) was initially used (mean 64.33, SD 13.12, range 38-80). In total, 13 individuals attended the experiment. One participant who showed up for the experiment was known to the experimenter and had their data subsequently excluded owing to likely bias. The final study sample thus comprised 12 participants (58% female; mean age, 19.75 years, SD 1.76 years; 67% Asian, 25% White, and 8% Mixed race).

### Measures

#### LSAS-SR

We used the self-report version of the LSAS-SR owing to its well-established validity and reliability in a large amount of previous literature [24]. The LSAS-SR allows for the classification of individuals into differing severity groups, as a higher overall LSAS-SR score is seen to correspond with greater social anxiety severity [24,31]. Furthermore, the LSAS-SR examines both affective aspects (ie, quantifying how anxious participants feel) using the fear subscale and behavioral aspects (ie, gauging to what extent they avoid various social situations) using the avoidance subscale. Each subscale consists of 24 items, with response items ranging on a 4-point scale from “none (0)” to “severe (3)” for the fear subscale, and “never (0)” to “usually (3)” for the Avoidance subscale. Prior studies indicate a high level of reliability of the LSAS-SR (Cronbach α=.95 [24]). In this study, the Cronbach α values for the fear and avoidance subscales were .69 and .69, respectively, with an overall Cronbach α of .83.

#### Social Phobia Screening Questionnaire

To cross-validate the LSAS-SR, we also used the Social Phobia Screening Questionnaire (SPSQ), which comprises 8 questions about how much fear individuals feel in various social situations, including speaking in front of a group of people, going to a party, and being alone with someone unfamiliar [32]. This measure has shown good validity in prior research [32]. It can be used with or without additional questions that allow an estimation of whether individuals reach the clinical cut-off for SAD and has been used in previous research to indicate subclinical social anxiety levels [33]. The response items ranged from “none (1)” and “some (2)” to “a lot (3)” (Cronbach α=.74).

### Ethics

The University Ethics Committee approved all the procedures and measures used in the study. Throughout the procedure, participants were reminded that their participation was voluntary and that they could withdraw their data at any time until used for statistical analysis. The collected data were anonymized and stored in a password-protected folder.

### Data Collection

The data were collected using the E4 Empatica research-grade multi-sensor wristband wearable. The device was selected as it simultaneously monitors various types of physiological data at predetermined sampling rates [34]. However, only HR, EDA, and ST data were explored in this study as they could be considered social anxiety markers [26-29]. E4 Empatica has not yet been used in many studies of this nature, although other multi-sensor wrist-worn wearables have demonstrated effectiveness [16,17].

**Figure 2:**
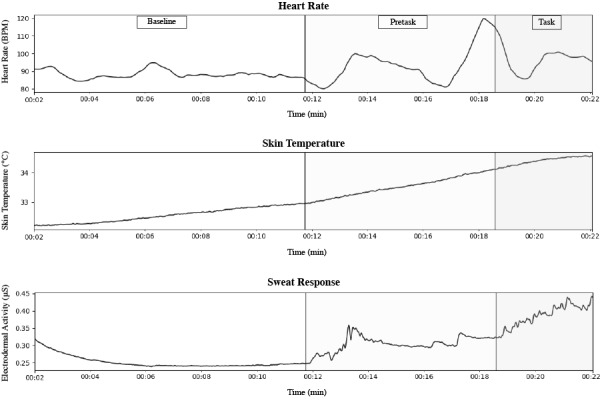
A participant’s physiological data sample during the experimental stages. BPM: beats per minute.

Using the default sampling rates of the E4 [35], EDA was measured in microSiemens (μS) at 4 Hz using stainless steel electrodes positioned on the inner side of the wrist. HR was measured in beats per minute (BPM) at 1 Hz using data derived from a photoplethysmography sensor. ST was measured in °C at 4 Hz using an infrared thermophile [35]. The data were collected throughout the duration of the experiment, an example of which is shown in Figure 2. The full data set and code needed to recreate the classification models and reproduce the results, as well as functions that enable further experimentation, is available in a designated GitHub repository (Multimedia Appendix 1).

### Experimental Protocol

**Figure 3:**
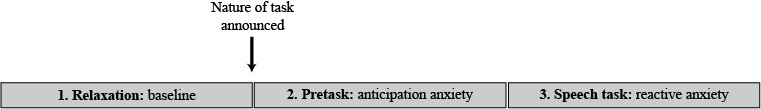
The stages of the impromptu speech task.

The experiments had an approximate duration of 30 minutes. Similar to previous studies [1,11,23], the experiment was split into 3 stages involving relaxation (baseline), task preparation (anticipation anxiety), and performance (reactive anxiety), as responses might differ across these stages [25]. Figure 3 illustrates the experimental stages. The timestamps for the stages were also recorded for labeling purposes. The experimental protocol is listed below.

First, the wearable was attached to the participant’s wrist. The procedure commenced with a 10-minute baseline period. During this time, participants were offered magazines and ocean sounds were played to create a calming effect.

Second, the nature of the task was then announced, and the participant was given 5 minutes to prepare a 3-minute speech on a selected subject from a choice of topics chosen on the basis of their anxiety-inducing potential. These included “*Is Brexit good or bad, and why?*,” “*Intelligence is not enough*,” and “*The history of Western Europe until the 2000s*.”

Third, a “judging” panel comprising experimenter confederates entered the room, and the participant performed the speech while being timed.

Finally, the participant was debriefed, and the wearable was removed.

### Data Preprocessing

The HR data were first upsampled to 4 Hz, similar to ST and EDA. A Moving Average Filter (Equation 1) was then applied to the data to remove noise [17] and reduce the risk of model overfitting [36,37].

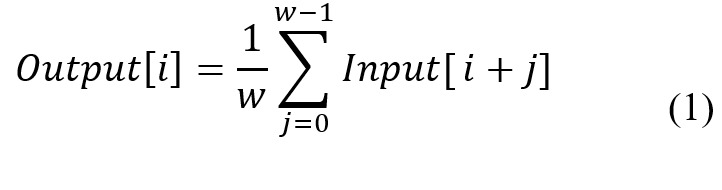



Where *w* refers to window size, Input[*i*] refers to original time series signal and Output[*i*] refers to processed time series signal.

**Figure 4:**
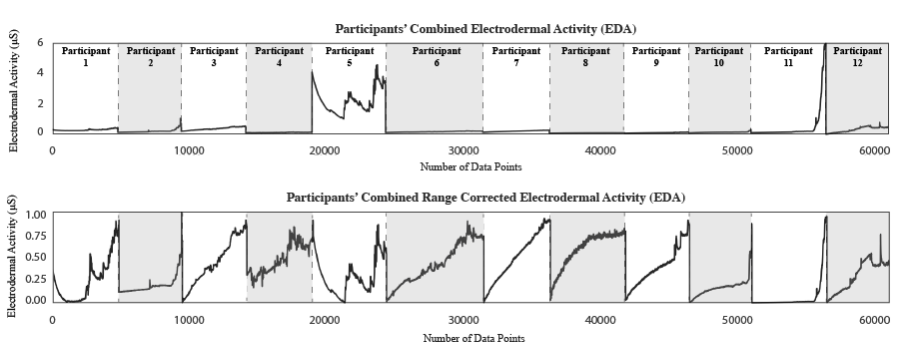
Participants' data before and after range correction.

An EDA range correction method (Equation 2) was applied to each participant’s EDA (*E*) data, see Figure 4 [38]. This removed inter-individual differences, particularly as physiological activation is believed to be better indicated by the variation within the EDA range rather than the range itself [39].

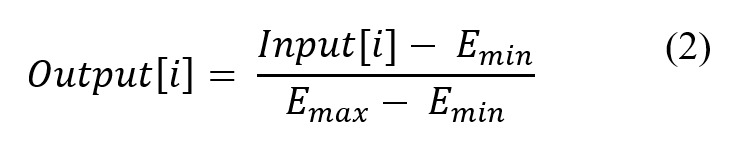



**Figure 5:**
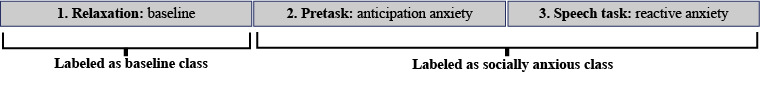
Labeling arrangement for classification investigation (1).

Following this, the labels were allocated on the basis of the experiment timestamps, assuming the suspected states were invoked. Classification investigation (1) examined whether models can be trained to classify baseline and socially anxious states. Therefore, the participants’ data were split into the respective classes and labeled using the experiment timestamps (Figure 5).

**Figure 6:**
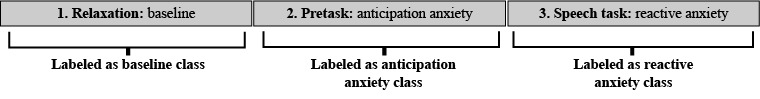
Labeling arrangement for classification investigation (2).

Classification investigation (2) focused on whether models can be trained to differentiate among baseline, anticipation anxiety, and reactive anxiety states. Therefore, the data were divided into the 3 respective classes using the timestamps and labeled as shown in Figure 6.

**Figure 7:**
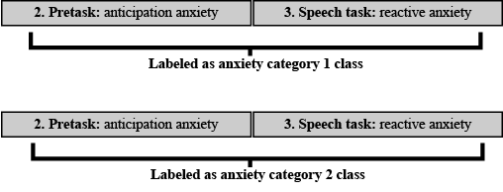
Labeling arrangement for classification investigation (3).

Finally, classification investigation (3) examined whether models could be trained to differentiate between anxiety experienced by individuals with differing levels of social anxiety. Therefore, the data were collected from participants within differing ranges of LSAS-SR scores, including anxiety category 1 (LSAS-SR:50-64) and anxiety category 2 (LSAS-SR:65-80) and was subsequently labeled (Figure 7).

The first 2 minutes from the baseline period were disregarded to account for acclimatization, and the recording was discarded after the task as it was not needed. All participant data were then combined.

The features were standardized to have zero mean and unit variance, which is a widely used scaling approach as algorithms such as Radial SVM assume features are centered around zero [36,40]. For each feature, the mean (µ) and the standard deviation (σ) were extracted from the raw training feature values. The training data were then standardized using equation (3), and the same transformation was applied to the test data [37].

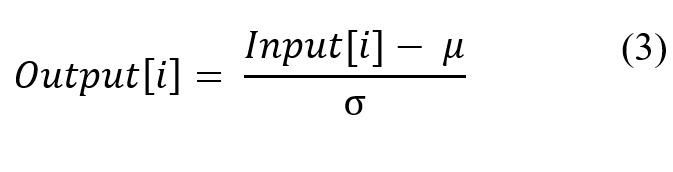



### Classification

The investigations were framed as supervised learning tasks owing to their classification nature. Four classification algorithms were explored: SVM, Random Forest, Decision Tree, and KNN. Furthermore, for classification investigation (2), a “*One Vs. Rest*” strategy was utilized as the investigation involved a multi-class data set.

The trained models were evaluated using 10-fold cross-validation. The method involves dividing the data set into k-folds with 1 fold for testing and the others for training. Confusion matrices were also utilized to calculate the average classification accuracy for each class.

## Results

### Study Descriptives

All study descriptives are shown in Table 2. In this sample, women had higher mean levels for all study variables than men (though the differences were nonsignificant, which is likely owing to the small sample). This is uncharacteristic, as women typically have a higher risk to develop anxiety and higher mean levels of social anxiety than men [41]. However, the self-reported LSAS scores were highly correlated with SPSQ scores (*r*=0.63; *P*=.05).

**Table 2:** Descriptives for all study variables by gender.

Gender		Participants, n	Score, mean (SD)
**Liebowitz Social Anxiety Scale fear subscale**			
	Women	7	1.3095 (0.33666)
	Men	5	1.5917 (0.11562)
**Liebowitz Social Anxiety Scale avoidance subscale**			
	Women	7	1.1845 (0.34766)
	Men	5	1.3583 (0.25786)
**Liebowitz Social Anxiety Scale avoidance subscale overall score**			
	Women	7	1.2470 (0.33190)
	Men	5	1.4750 (0.15548)
**Social Phobia Screening Questionnaire**			
	Women	7	1.3469 (0.36288)
	Men	5	1.7429 (0.29277)

### Combined Modalities

For classification investigation (1), the yielded accuracies were between 97.54% and 99.48%, as shown in Table 3. For investigation (2), the accuracies were between 95.18% and 98.10%, as shown in Table 4. Additionally, for investigation (3) the yielded accuracies were between 98.86% and 99.52%, as shown in Table 5. In each classification investigation, Radial SVM outperformed other classifiers (Tables 3-5).

**Table 3:** Cross-validation results for classification investigation (1).

Classifier	Overall performance, %	Baseline state accuracy, %	Social anxiety state accuracy, %
Radial Support Vector Machine	99.48	99.40	99.52
K-Nearest Neighbours	99.08	99.12	99.05
Decision Tree	97.54	99.04	96.59
Random Forest	97.96	99.38	97.13

**Table 4:** Cross-validation results for classification investigation (2).

Classifier	Overall performance, %	Baseline state accuracy, %	Anticipation anxiety state accuracy, %	Reactive anxiety state accuracy, %
Radial Support Vector Machine	98.10	99.30	98.37	95.52
K-Nearest Neighbours	97.61	98.99	97.28	95.78
Decision Tree	96.63	99.39	96.86	91.36
Random Forest	95.18	99.27	95.99	85.99

**Table 5:** Cross-validation results for classification investigation (3).

Classifier	Overall performance, %	Anxiety category 1 accuracy, %	Anxiety category 2 accuracy, %
Radial Support Vector Machine	99.52	100	99.03
K-Nearest Neighbours	98.86	99.35	98.36
Decision Tree	99.04	100	98.09
Random Forest	99.34	100	98.70

**Figure 8:**
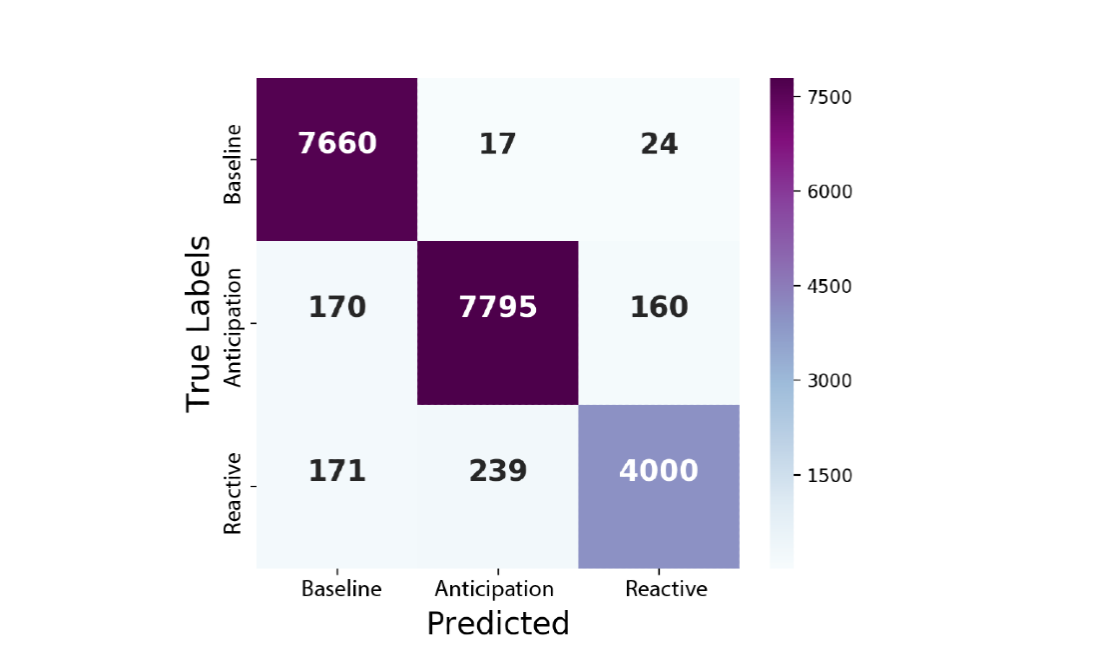
Confusion matrix from classification investigation (2) using the Decision Tree.

There were common class misclassification patterns among all classifiers. For investigation (1), the models were less able to classify anxious states (Table 3). For investigation (2), reactive anxiety was misclassified the most and often mistaken for anticipation anxiety (Figure 8). Additionally, in investigation (2), the baseline class was most accurately classified, as shown in Table 4. For investigation (3), the models were not as effective at classifying anxiety category 2 (Table 5).

### Singular Modalities

**Figure 9:**
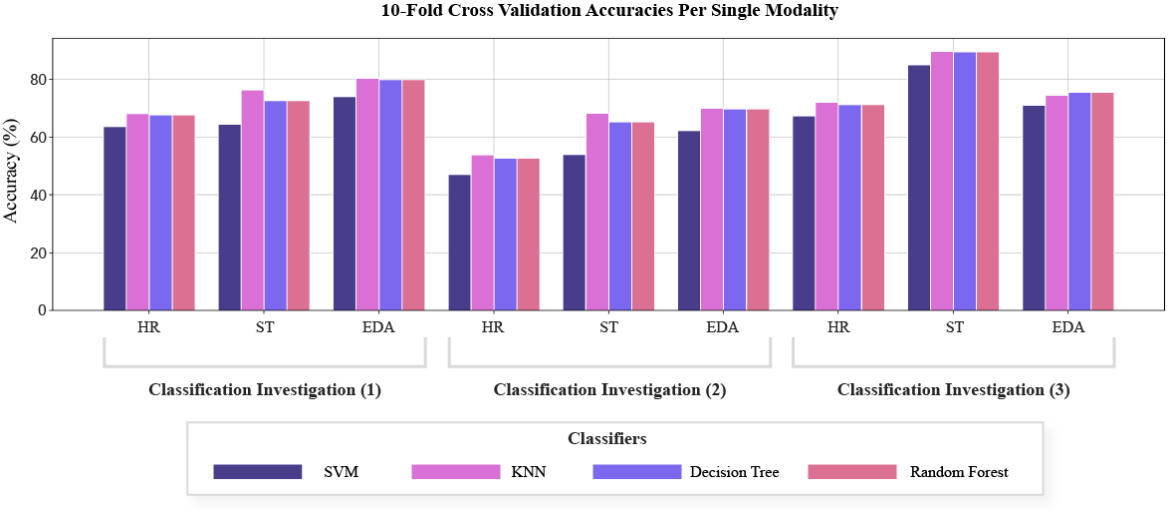
Accuracies per modality. EDA: electrodermal activity, HR: heart reate, KNN:K-Nearest Neighbours, ST: skin temperature, SVM: Support Vector Machine.

The singular modality results are shown in Table 6 and Figure 9. In classification investigation (1) EDA yielded 80.46% and was shown to have the highest predictive capability. EDA was also shown to have the highest classification accuracy of 70.02% for investigation (2), whereas ST was the most effective modality for investigation (3) with an accuracy of 89.47%. For each classification investigation, HR was observed to be the least effective modality. Furthermore, KNN generally outperformed other classifiers (Table 6).

**Table 6:** Highest cross-validation results per single modality.

Modality			Classifier with the highest performance		Overall performance, %
**Classification investigation 1**					
	Heart rate	K-Nearest Neighbours		68.18	
	Skin temperature	K-Nearest Neighbours		76.30	
	Electrodermal activity	K-Nearest Neighbours		80.46	
**Classification investigation 2**					
	Heart rate	K-Nearest Neighbours		53.91	
	Skin temperature	K-Nearest Neighbours		68.32	
	Electrodermal activity	K-Nearest Neighbours		70.02	
**Classification investigation 3**					
	Heart rate	K-Nearest Neighbours		72.00	
	Skin temperature	K-Nearest Neighbours		89.47	
	Electrodermal activity	Random Forest and Decision Tree		75.66	

## Discussion

### Principal Findings

#### Combined Modalities

This study aimed to determine if ML models could be trained to (1) classify baseline and socially anxious states, (2) differentiate among baseline, anticipation anxiety, and reactive anxiety states, and (3) classify social anxiety with differing severity levels of social anxiety. High accuracies were obtained when differentiating between baseline and socially anxious states, suggesting that it is possible to detect social anxiety using HR, ST, and EDA. These high accuracies are likely due to physiological differences between baseline and socially anxious states and have also been shown in previous research [10-12].

The models also yielded high accuracies when classifying among baseline, anticipatory, and reactive states. The classifiers’ ability to differentiate between reactive and anticipatory anxiety might be due to the varying responses during these stages. It is, therefore, likely possible to detect the nature of social anxiety experienced on an individual basis.

The models also yielded high accuracies when differentiating between marked and moderate social anxiety. This demonstrates the possibility to identify social anxiety levels using physiological indices, implying that individuals with differing severity levels of social anxiety exhibit diverse physiological responses. This is in line with prior research indicating that individuals with greater social anxiety exhibit responses consistent with greater threat [10].

The results also indicated that higher modeling accuracies were yielded when all modalities were combined [42]. Research shows that models created using singular modalities may be underfit owing to lack of data [37]. This is likely because each physiological index contains varying information that enables classifiers to differentiate among certain classes, thus providing measurement granularity. Furthermore, when modalities were combined, Radial SVM outperformed the other classifiers in all investigations, which is possibly owing to the classifier’s ability to formulate complex decision boundaries [37,43].

Finally, certain classes were commonly misclassified, which could be explained by class imbalances owing to the different durations of each stage during the data collection sessions. Class imbalances can cause classifiers to bias toward larger classes [44].

#### Singular Modalities

Each modality had varying predictive capabilities, despite the complexity of the physiological indicators used in the study. EDA was the most effective singular modality when differentiating between baseline and social anxiety states (including anticipatory and reactive states). This is possibly because EDA comprises the sum of phasic and tonic components that change following stimuli, which is likely because sweat glands responsible for EDA variation are entirely controlled by the SNS, whereas HR and ST are mediated by both the PNS and SNS [26,27,29]. Thus, EDA represents an accumulation of information that could indicate social anxiety [27].

ST was the most effective modality when differentiating between anxiety experienced by individuals with differing severity levels of social anxiety. This might be because individuals with greater social anxiety exhibit differing amounts of blood flow to the skin. This surprising finding highlights the predictive capability of ST collected around the wrist and suggests that it could be viewed as a novel social anxiety marker.

HR showed the lowest effectiveness in all investigations, which might be explained by HR being mediated by the PNS and SNS [26,27]. The comparatively low recognition accuracies may also be a result of HR being sampled at the lowest rate.

Furthermore, KNN was the most effective classifier when the modalities were singular, which is likely because KNN can formulate complex decision boundaries between classes.

### Limitations

Despite these promising results, these findings are preliminary. The sample size was small, with the COVID-19 pandemic preventing further data collection. Prior to the COVID-19 pandemic, we intended to collect test data in “real-world” settings to evaluate the models’ ability to detect social anxiety in practice. Instead, the models were evaluated using a subset of data from the experiment. Although this approach is often used in ML studies [16,17], it does not offer a realistic indication of model generalizability. Therefore, given the small sample size, our results need to be interpreted cautiously.

Additionally, classifiers may have been biased toward certain classes owing to the moderately differing class sizes (Multimedia Appendix 2). This may have accounted for the high accuracies but reduced model generalizability [44]. Like other studies of a similar nature (such as affect recognition studies using physiological data [45]), it was difficult to establish the ground truth of the data with respect to the presence and nature of social anxiety. Therefore, labeling was assumed to be aligned with the experimental protocol.

Furthermore, the physiological responses from the individuals could have been influenced by external factors such as caffeine and alcohol consumption [46,47], though this was not mitigated in the current study design. It is also important to note that EDA measurements can be affected by environmental conditions such as humidity and room temperature [27]. Although the experiments took place in the same room, these variables were not monitored and controlled. In sum, all of these limitations remain challenges for future research.

### Comparison With Prior Work

Despite its limitations, this study has extended previous work and applications focusing on supervised machine learning in the field of physiological anxiety detection. This experiment was informed by existing study protocols, such as using an impromptu speech task, which is a cornerstone of experimental work invoking social anxiety [11,22,23]. Additionally, the study utilized the LSAS-SR measure, which is a widely used measure demonstrating good psychometric properties in previous research [24], and the social anxiety self-reports were cross-validated using another well-known indicator of subclinical social anxiety (SPSQ [32]). Overall, our findings also align with those of prior studies indicating that EDA is a “directed and undiluted” representation of the SNS [27]. Although prior work has focused on EDA as an indicator, physiological measurement from the anatomical site of the wrist had not been explored in a social anxiety context.

### Conclusions

This study examined whether social anxiety could be detected in young adults using physiological data (HR, ST, and EDA) from wrist-worn sensors. The findings indicate that it is possible to detect social anxiety and its severity using this approach. Future work in this area has the potential to identify novel methods of detecting and monitoring subclinical social anxiety in young adults, which could help counteract development into SAD. As mental health provision is transitioning toward digital interventions, it is crucial that they are evidence-based and can target individuals with subclinical levels of social anxiety. The ability for future interventions to detect social anxiety before it escalates further could have great social and economic benefits for health care, society and those who experience its consequences.

## Appendix

Multimedia Appendix 1 A Github repository was created to accompany this work. The repository contains the full dataset and code needed to recreate the classification models and reproduce the results, as well as functions that enable further experimentation.

Multimedia Appendix 2 The following tables illustrate the class data distributions for each classification investigation.

## Abbreviations

ANS: autonomic nervous system
BPM: beats per minute
EDA: electrodermal activity
HR: heart rate
KNN: K-Nearest Neighbours
LSAS-SR: self-reported version of the Liebowitz Social Anxiety Scale
ML: machine learning
PNS: parasympathetic nervous system
SAD: social anxiety disorder
SNS: sympathetic nervous system
SPSQ: Social Phobia Screening Questionnaire
ST: skin temperature
SVM: Support Vector Machine
