# Supplementary material for: Detecting Subclinical Social Anxiety Using Physiological Data From a Wrist-Worn Wearable: Small-Scale Feasibility Study
Source: JMIR Form Res. 2021 Oct 7;5(10):e32656. doi: 10.2196/32656 (PMC8532020; doi:10.2196/32656)
Supplement: Multimedia Appendix 1 [file formative_v5i10e32656_app1.docx]

<https://github.com/rs2416/Detecting_Social_Anxiety>
