# Supplementary material for: Detecting Subclinical Social Anxiety Using Physiological Data From a Wrist-Worn Wearable: Small-Scale Feasibility Study
Source: JMIR Form Res. 2021 Oct 7;5(10):e32656. doi: 10.2196/32656 (PMC8532020; doi:10.2196/32656)
Supplement: Multimedia Appendix 2 [file formative_v5i10e32656_app2.docx]

Table 7. Classification investigation (1) class data distribution

|  | Baseline State Class | Social Anxiety State Class |
| --- | --- | --- |
| Percentage of Total Samples (%) | 38.65 | 61.35 |
| Number of Data Points | 23584 | 37428 |

Table 8. Classification investigation (2) class data distribution

|  | Baseline State Class | Anticipation Anxiety State Class | Reactivity Anxiety State Class |
| --- | --- | --- | --- |
| Percentage of Total Samples (%) | 38.35 | 39.91 | 21.74 |
| Number of Data Points | 23282 | 24228 | 13200 |

Table 9. Classification investigation (3) class distribution

|  | Anxiety Category 1 Class | Anxiety Category 2 State Class |
| --- | --- | --- |
| Percentage of Total Samples (%) | 50.00 | 50.00 |
| Number of Data Points | 10002 | 10000 |
